# On the Role of Digital Twins in Data Spaces

**DOI:** 10.3390/s23177601

**Published:** 2023-09-01

**Authors:** Friedrich Volz, Gerhard Sutschet, Ljiljana Stojanovic, Thomas Usländer

**Affiliations:** Fraunhofer IOSB, 76131 Karlsruhe, Germany; gerhard.sutschet@iosb.fraunhofer.de (G.S.); ljiljana.stojanovic@iosb.fraunhofer.de (L.S.); thomas.uslaender@iosb.fraunhofer.de (T.U.)

**Keywords:** data space, digital twin, asset administration shell (aas), industry 4.0

## Abstract

Industry 4.0 supports the vision of networked machines in decentralized production plants across the value chain. Hence, it requires highly connected partners exchanging relevant data about products, processes, and production resources. This paper proposes the usage of data spaces and digital twins to enable this Industry 4.0 vision and investigates the building blocks to realize a data space for Industry 4.0, e.g., the integration of digital twins inside the data space based upon the latest specification of the Industry 4.0 Asset Administration Shell. A prototypical implementation shows the feasibility of storing product carbon footprints inside a digital twin and sharing it over a data space with other partners.

## 1. Introduction

The transition toward a circular economy is a pressing challenge addressed by the European Green Deal, and Industry 4.0 is contributing by enabling the digital transformation of the industry toward more sustainable and efficient practices [1].

Standardized digital twins play a crucial role in Industry 4.0 by enabling the seamless integration of different components in a factory or industrial plant [2]. By providing a standardized information model, digital twins allow machines and software systems to communicate with each other in common languages and models, regardless of their manufacturer or technology. Plattform Industrie 4.0 introduced the Asset Administration Shell (AAS) as their solution of a standardized digital twin [3]. However, the specification does not yet address data sharing according to specified data sovereignty policies. The International Data Spaces Association (IDSA) offers a reference architecture for data spaces called the International Data Spaces Reference Architecture Model (IDS-RAM). It describes principles of data sharing including data usage policies and provides an information model for data asset descriptions, as well as a standardized exchange API. Furthermore, the IDS community offers several open-source reference implementations of the IDS-RAM [4].

This paper explores the potential of combining digital twins with data spaces to enable data-sovereign sharing with digital twins as the data sources and data sinks. For this, the current state of the art regarding digital twins in data spaces is analyzed. The possible role of digital twins in different data space components is discussed, and an approach for integration based on the IDS and AAS is described. The focus of this approach is to enable end-users to benefit from both digital twins and data space while reducing barriers to entry due to complexity and onboarding effort.

In Section 2, we provide an overview on how data spaces are designed and how to realize digital twins with standards. In Section 3, we explain the IDS components and discuss the role of digital twins inside the IDS. In Section 4, we present our approach and implementation for integrating the AAS with the IDS in a user-friendly environment. In Section 5, we compare our approach to the state of the art. In Section 6, we summarize the work and explain the required future work.

## 2. Background

### 2.1. Definition and Design of Data Spaces

The EU-funded OPEN DEI project recently released a position paper to define design principles for data spaces and their building blocks [5], involving more than 25 organizations. The authors of [6,7] dealt with the topic of domain independent data space design. In [8,9], several data spaces for different domain specific application are discussed. The OPEN DEI position paper is based on the design approaches and components proposed in the current research, including but not limited to these books and papers. It aligns the terms and definitions of a data space from a domain-independent perspective.

**Figure 1:**
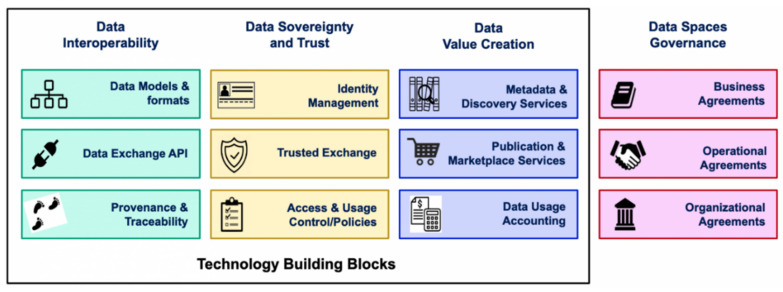
Data space building blocks by OPEN DEI [5].

Specifically, a data space is a “federated data ecosystem within a certain application domain based on shared policies and rules” [5]. The access and usage rights are granted by those who are entitled to publish this data. Data spaces comprise building blocks that are divided into technical and governance building blocks. These building blocks enable interoperability, trust, data value creation, and governance within the data space [5]. Figure 1 shows the OPEN DEI project definition of building blocks.

The technical building blocks are divided into nine building blocks. Data spaces can range from a minimal data space [10], which only fulfills basic features such as secure data transfer, to whole data space networks including marketplaces and additional platform services.

Most of these building blocks are designed and implemented in such a way that they are not specific to a certain application domain or use-case. However, the technical building block involving data models and formats is highly specific and needs to be adapted for all participants in the domain, namely, Industry 4.0 (see Section 3.2.1). Additionally, organizational challenges arise since all stakeholders need to agree on the decentralized architecture and implement it in their infrastructure. The governance building blocks consist of such organizational, business, and operational agreements.

In the remainder of this section, we briefly describe the main functionalities of the verticals used to organize the data space building blocks.

#### 2.1.1. Interoperability Building Blocks

To enable interoperable exchange for participants in a data space, standards and specifications for data models and data formats are required. It is necessary to agree on the models that will be used for the data, since the meaning and semantics of the data should be understood by all participants. The formats or models can be chosen freely, but agreeing on the least possible subset of formats and models minimizes the need to convert and map between them.

The next interoperability building block is the data exchange API. For uniform, interoperable data exchange, the participants in a data space need to agree on the messages and protocols to be used between company borders. The protocols can be defined freely, but to achieve high interoperability even across data space domains, reference architectures such as the IDS with its communication patterns should be considered. Additionally, technical implementations [5] to realize this communication are required.

The last building block, provenance and traceability, describes the ability to trace the data path along different data space connectors and participants. For this, the connectors usually log data transfers and store them in a central data space component available to all participants. This enables traceability of data, even if it has left the connector of the participant.

#### 2.1.2. Data Sovereignty and Trust Building Blocks

Identity management in a data space is an important building block to create trust and security, so that each participant is really who they claim to be. Especially when exchanging or providing environmental indicators such as product and production emissions, trust between participants needs to be established. Each participant in the data space requires a unique, verifiable identity which is usually provided by components such as an identity provider through identity certificates [4].

In the case of trusted exchange, data space participants must decide which components are considered as trusted and how trust between unknown participants can be achieved. Data space initiatives such as the IDS usually verify their building block components such as connectors and identity providers by third parties to guarantee a high level of security and prevent security flaws during technical implementation [11]. Additionally, an identity is only provided to trustworthy participants.

Access control is concerned with granting and restricting access to data resources [4]. Usage control is an extension to access control that attaches additional obligations to the data usage with usage policies. In data spaces, technical enforcement of such access and usage rules would allow to govern the way in which published resources are used by which participants. While access and usage control are important for owners of sensitive data, technical enforcement of the usage rights requires complex systems. In most cases, the usage policies are attached to data resources, and the other party is responsible to adhere to these policies. Typically, technical enforcement of usage policies can only be achieved in a limited manner. In practice, it must be complemented by legal enforcement.

#### 2.1.3. Data Value Creation Building Blocks

The metadata and discovery services building block is mostly concerned with enabling participants to find each other and the corresponding data through a protocol. For the metadata, data resources must be described with an information model, for example, the IDS information model [4]. Otherwise, the data space participants will not be able to understand their data descriptions. For the discovery, participants want to query a central component such as an IDS metadata broker [12] to discover suitable data sources.

The publication and marketplace services building block is concerned with the publication of data offerings and marketplace services enabled by data sharing. The previously mentioned IDS metadata broker publishes the metadata of participants in the data space. An example for marketplace services in the Industry 4.0 would be manufacturing as a service (see [13]).

Data usage accounting describes the monitoring of data usage in the data space. For example, providers of battery data can monitor the usage and even charge their customers for the usage of data.

#### 2.1.4. Additional Technical Building Blocks

The OPEN DEI project also mentions more technical building blocks to facilitate connection of additional systems into the data space but, because of their edge location, does not include them in the shown figure [5], i.e., the system adaptation building block to facilitate the transfer of data to and from participants’ systems. The data processing building block includes systems connected to the data space via systems adapters, which process shared data. The data visualization building block provides data representation and visualization features for shared data. The OPEN DEI project states that the list of technical building blocks is not exhaustive and does not dictate a specific technical implementation [5].

#### 2.1.5. Governance Building Blocks

The governance building blocks are not technical building blocks, but contain business, operational and organizational agreements between the data space participants. Here, the service-level agreements (SLAs) between companies are defined, which are contracts between a company providing data services and a company using these services. The data space participants also need to agree on the functional, technical, operational, and legal aspects of the data space.

In Section 3, we identify building blocks relevant for Industry 4.0, discuss challenges to their realization with digital twins, and describe existing results.

### 2.2. Digital Twins

In recent years, digital twins (DTs) technology has gained more and more importance for many domains and applications. The basic concepts of DTs were developed in the 1990s, where dynamical models of real-world components and processes were used for diagnosis of technical systems. The models (ok-models and fault-models) used were linked to sensors in physical systems to monitor the real-world and simulate the future behavior. In case of discrepancies of real-world and simulated behavior, the kinds of discrepancies were used to start a diagnosis of the technical system [14].

Manufacturing use cases were solved with DTs in the 2000s. An example is the “production assistant” developed for the automotive industry by Fraunhofer IOSB [15]. This DT represented one component of a production control system and was able to signal production problems for the next two shifts of an assembly shop. Therefore, simplified models of manufacturing plants were periodically updated with production data from the real-world factory. The future behavior of the plant was determined by an event-based simulation.

Although these basic concepts originated about 30 years ago, there are many heterogeneous definitions for DTs. As stated in [16], functionalities of DTs vary. The literature review shows that the research is still in its infancy, and significant differences in the maturity of DT exist.

The most general definition is given by the Industrial Digital Twin Association (IDTA): “A digital twin is a virtual representation of real-world entities and processes, synchronized at a specified frequency and fidelity”.

There are different types of DTs according to [17]: (i) digital twin prototypes, which are built before the physical product exists. They are used for designs, analysis and processes to realize the physical product; (ii) digital twin instances, which are linked with the physical counterpart; (iii) digital twin aggregates, which represent data aggregations across multiple instances for the purpose of prognostics and learning.

The authors in [16] characterized several applications as digital shadows instead of digital twins, which are defined by “an information exchange from the physical to the virtual component but not vice versa”.

DTs should support the whole product lifecycle of an asset or product from conception through design and development, to deployment and maintenance, and finally to its decomposition. DTs are able to interact using standardize interaction methods. They are accompanied by a standard definition of its term and taxonomy.

The AAS is developed by Plattform Industrie 4.0 as a conceptual consequence of the Reference Architecture Model Industry 4.0. According to the working group 1 (WG1), “Reference Architectures, Standards and Norms”, an AAS is a “standardized digital representation of the asset, and a corner stone of the interoperability between the applications managing the manufacturing systems. It identifies the administration shell and the assets represented by it, holds digital models of various aspects (submodels), and describes technical functionality exposed by the administration shell or respective assets” [3] (Details of the AAS, Part I). The AAS is used to implement DTs for Industrie 4.0. The standardized way ensures interoperability of DTs among different suppliers. The AAS is a digital representation of an asset. Ideally, it represents all the information and functionalities of an asset. This includes characteristics, properties, parameters, measurement data, and the capabilities of an asset. An AAS consists of several submodels describing detailed properties of the asset. The form of these submodels is standardized through submodel templates. The standardization is organized by the IDTA [2]. Different communication channels can be used to link the AAS to the physical asset it represents.

The AAS standard is described in different specification parts, for example, the metamodel and serialization of the AAS (part 1) and communication/interaction with AAS instances at runtime (part 2). All parts are under further development.

In addition to the AAS standard, there are several competing standards which can partly be mapped on each other as shown by [18]. The most prominent competing standards are described below.

- The Digital Twins Definition Language (DTDL) was developed for the Azure platform by Microsoft [19]. The specification of DTDL defines it as “a language, to describe models of IoT plug and play devices, DTs of devices, and logical DTs”.
- The Next-Generation Services Interface-Linked Data (NGSI-LD) standard was published by the Context Information Management (CIM) of the European Telecommunications Standards Institute (ETSI) Industry Specification Group (ISG) [20]. It “… enables users to provide, consume, and manage context information in a variety of scenarios and involving multiple actors”.
- The Eclipse Foundation developed another standard for DTs called Eclipse Vorto [21]. The main purpose of Eclipse Vorto is to ease the communication of different IoT devices by providing a normalized API for easy integration into software solution.
- The Web of Things (WoT) Things Description (TD) standard was published by the WoT Working Group of the World Wide Web Consortium (W3C). The Things Description was described in [22]: “A Things Description describes the metadata and interfaces of a Thing. A Thing is an abstraction of a physical or virtual entity that enables and participates in interactions with the WoT”.

According to the Plattform Industrie 4.0, the AAS standard is the realization for DTs in the Industry 4.0. We note that this is the only specification targeting Industry 4.0. Because of this, our integration targets the AAS and IDS specification.

Currently, there are several implementations of the AAS. The authors of [18] provided a survey and an evaluation of four open-source implementations that are currently to be considered. The paragraphs below are summaries from this paper.

The AASX Server [23] is being developed in the context of the IDTA. Its code is based on the AASX Package Explorer, which is the most prominent modeling tool for the AAS. AASX Server is implemented in C#. NET.

Originating from the BaSys 4.0 and the follow-up BaSys4.2 research projects funded by the German Federal Ministry of Education and Research (BMBF), Eclipse BaSyx [24] provides an implementation of the reactive AAS (Type 2). Eclipse BaSyx provides a feature-rich ecosystem including a client SDK, as well as components for asset integration and AAS visualization.

FA³ST Service [25] is being developed at Fraunhofer IOSB as part of the Fraunhofer Advanced Asset Administration Shell Tools for Digital Twins (FA³ST), a collection of tools for modeling, creating, and using DTs based on the AAS specification.

The NOVA Asset Administration Shell (NOVAAS) [26] is being developed by NOVA School of Science and Technology in the context of the H2020 PROPHESY project. The implementation follows a no/low-code approach based on Node-RED, a flow-based programming tool.

The main conclusion of the work reported in [18] is that there is no AAS implementation that fully implements the AAS specification. There are some aspects of the AAS specification that are not covered by any implementation, and many that are not fully implemented. However, all considered AAS implementations support the minimum required functions.

In addition, the survey [18] provides useful feedback to further refine the AAS specification to help software developers understand the semantics of the AAS metamodel and API.

### 2.3. Integration of Digital Twins into Data Spaces

While data sharing can be conducted solely with digital twins, e.g., AAS communication with AAS API, most DT specifications do not deal with data-sovereign sharing to the same extent as the IDS. Firstly, specifications such as the AAS only briefly address security, e.g., token-based authentication. Secondly, the topic of data policies and usage contracts are not addressed by digital twins.

Data spaces and digital twins are two distinct concepts, but they can complement each other to enable interoperable, secure, and standardized data exchange within industrial ecosystems. In [8], the authors argued that digital twins and data spaces are inextricably linked, and a system approach is required to link both concepts. Both concepts aim to combine datasets in a suitable way. The authors explained that the digital twin idea originated from managing lifecycle data of products. Since these data are acquired for a DT, it can be used in later lifecycle stages such as maintenance or recycling [8]. Therefore, we argue that Industry 4.0 platforms require both digital twins and data space components.

Although the topic is very important, there are few papers on integration of the different initiatives, namely, the IDS and the AAS. In Section 5, we compare our approach to the state of the art and point out differences.

## 3. Data Space Based on Digital Twins

In this section, we present the IDS and map their components to the building blocks previously mentioned. This allows us to recognize DT-relevant building blocks and discuss several design choices for a data space based on digital twins. The IDS is one possibility to realize data spaces and was selected because it offers the most reference implementations of components. Similar solutions could target the GAIA-X architecture [27].

### 3.1. International Data Spaces

**Figure 2:**
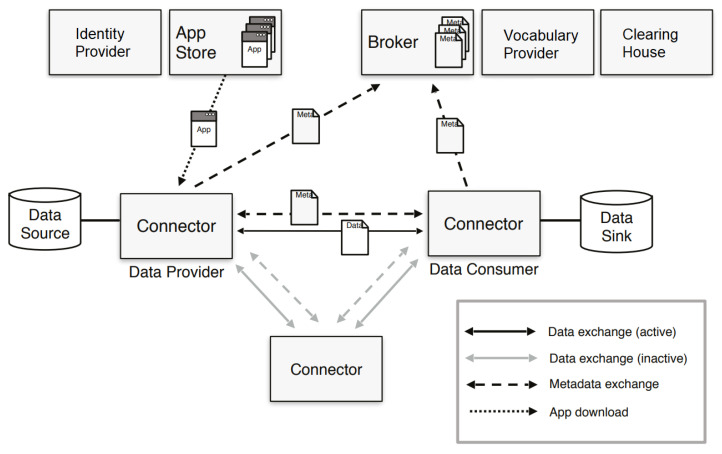
Data space by IDSA. Reproduced with permission from [4].

Figure 2 shows the data space view shared by the IDSA. It addresses nearly all previously described building blocks except for marketplace services, which are usually built on top existing components.

A central component required for data providers and consumers is the connector, which is the gateway of an organization to participate in the data space. It is part of several technical building blocks (see Table 1). Most notably, the connector is used to transfer data and conduct data policy negotiations.

The identity provider provides a unique identity for each connector. It is part of the identity management building block in the trust building blocks. The connectors store the identity and initiate verification.

A broker is a component where all connectors can register to publish their data offerings to a broader audience. It is also the component used to search for suitable data offerings. This component is part of the data value building blocks; specifically, it uses the metadata and discovery protocol building block and is part of the publication services.

Vocabularies and ontologies are usually provided by a component called the vocabulary provider. Here, the semantic meanings can be requested, which are used in the descriptions of data offerings. They support the publication services but are inherently part of the data models building block.

The clearing house logs all data transactions taking place to provide traceability and provenance of data in the network. It also supports the data usage accounting building block to create invoices for data usage.

An app store is a component to provide additional applications for connectors, e.g., an application to offer emission permits based on the current process carbon footprint. With these applications, additional functionality can be introduced into the connectors if the connector implementation supports installation of IDS apps.

A central component required for data providers and consumers is the connector, which is the gateway of an organization to participate in the data space. It is part of the interoperability building blocks, namely, the data exchange API.

Table 1 shows an overview of the components and the building block (introduced in Section 2.1) they contribute to. The table confirms that all data space building blocks can be realized with IDS components.

### 3.2. Role of Digital Twins in Data Spaces

This section clarifies the role of DTs in data spaces. For this, DT-relevant data space building blocks are selected, and the integration between DT and data space is explained. Additionally, DT and data space components are compared.

#### 3.2.1. DT-Relevant Data Space Building Blocks

**Figure 3:**
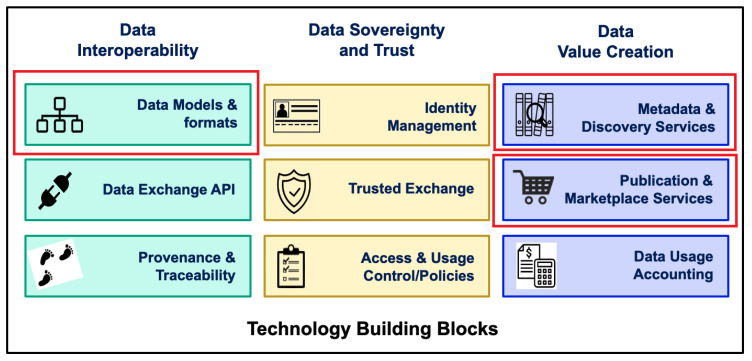
DTs in data space building blocks.

Figure 3 shows the building blocks of the data space that need to be considered when releasing a data space based on DTs. DTs are part of the interoperability building blocks, more precisely, the data models and formats building block, as shown in Figure 3. DTs enable interoperable exchange between participants by defining a subset of suitable data formats and integrating data models. The AAS relies on other domain-specific ontologies and vocabularies but harmonizes the API and integration of data sources into the data space. As such, the data formats and models are clear for all participants of the data space.

Additionally, they must be considered when describing the metadata and catalogues in the data space; otherwise, the data space participants would not be able to find the correct DT. In general, the goal of the metadata and discovery services building block is to enable data space participants to find suitable data sources (DTs).

DTs can also be reflected in marketplace services, e.g., enabling manufacturing as a service, where the capabilities of a machine are offered to all data space participants. These services depend on the business case of the data space.

Other building blocks are not DT-specific since they facilitate data space functionality in a generic way.

The remainder of this section discusses the challenges and solutions to realizing the relevant building blocks mentioned above.

#### 3.2.2. DTs and IDS Connectors

Plattform Industrie 4.0 proposes usage of the AAS (see Section 2.2) as a standard for realizing DTs. In the case of the use of an AAS as a data source, it provides a uniform application programming interface (API), as well as a set of defined communication protocols, and can itself synchronize several data sources into a logical unit like a digital product passport (DPP). In this case, the DT of a product can provide a DPP, but the two terms are not synonyms since a DT can provide additional functionality such as simulation or data-driven services. The ZVEI e.V. proposes the AAS as implementation of a DPP [28].

There are several other specifications to realize standardized DTs [18]. The possibility to reference other ontologies or models in the AAS and the submodel templates, which themselves are models to describe common aspects like a carbon footprint, are advantages in the realization of a DT. We propose the usage of the AAS to describe products, processes, and humans inside a company while leveraging data space building blocks to share the AAS as a data source across company borders. In our view, the AAS is also the most promising solution to realize DPPs, but different solutions might emerge as seen in [29].

**Figure 4:**
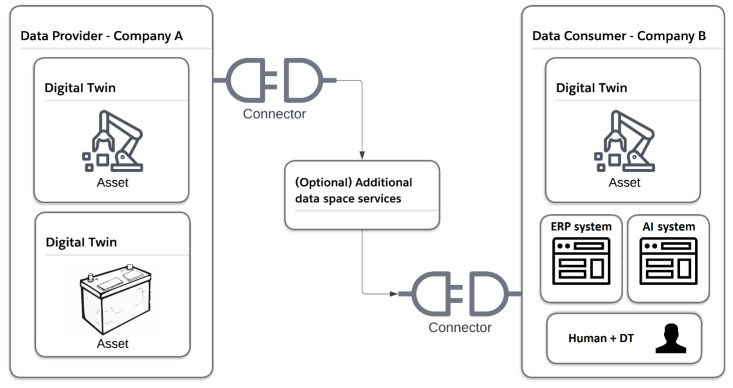
Combination of DT and DS (simplified).

DTs and IDS connectors can both be deployed inside a company or on cloud infrastructure and used independently. However, DTs usually represent a physical asset found in the company while IDS connectors are gateways to data spaces across company borders. As such, we suggest the combination of both concepts as shown simplified in Figure 4.

In most cases, IDS connectors initiate data transfers with other IDS connectors over Hyper Text Transfer Protocol Secure (HTTPS) [7]. However, there are also specific data space protocols available or in development, such as the IDS Communication Protocol (IDSCP) and Dataspace Protocol [30,31]. In the case of HTTPS, both metadata and payload are exchanged between connectors.

The AAS specification chose two communication protocols (HTTPS, OPC UA) for its API [18], which can be leveraged by IDS connectors for data integration. By describing AAS HTTPS endpoints in the connector metadata, the data are requested from the AAS over HTTPS (AAS API) and sent to the other participant over HTTPS (IDS API).

If the data source is not an HTTPS endpoint, e.g., an OPC UA machine server or AAS without HTTPS, the connector must support this communication protocol through its backend data services. By using the AAS as a data source in IDS connectors, the integration into the dataspace is simplified by focusing on the subset of protocols the AAS specified. In this case, the AAS is responsible for the synchronization between the AAS and physical asset, process or human.

Below, we present available connectors and their technology readiness to select suitable solutions. The open-source Eclipse Dataspace Connector (EDC) is a technical implementation for communication between participants in a data space with an open community behind it [32]. Different connectors offer specific features, e.g., the open-source Trusted Engineering (TRUE) connector [33] supports technical enforcement of usage rules. Connectors can communicate with other connector implementations that implement the same communication pattern.

While other data space architectures [4] propose the transfer of data through the connector itself, the Eclipse Foundation proposes a split data and control plane. This means, that, after the connector verifies the data transfer in the control plane, the data are sent from the data source to the data sink via a split connector data plane. The data transfer is initiated by the connector, which means that the data source needs to support the data plane suggested by the connector. If the connector has a unified control and data plane, the data are transferred directly between the connectors. In this case, the connector requests the data directly from the data source and transfers it to the other connector.

Since the EDC has a split control and data plane while the TRUE connector follows the unified planes approach [4] more closely, they are currently not compatible. This issue will be solved in the H2020 project that also partly funds this paper.

In a data space, the connector provides a catalogue of available data sources to other connectors. These data sources can be anything from databases, ERP systems, and files to web services. In all cases, the integration of data sources into the connector or data plane is a technical challenge, which usually requires manual effort. Since there are numerous communication protocols and data formats offered by data sources, we propose the usage of standardized DTs as data sources.

DTs can be deployed on the edge to realize a synchronization between the digital and physical entity in a company. However, they can also be deployed in a centralized cloud to collect public data sources and process data. It is important to note that DTs do not primarily focus on data sovereign sharing. As such, the security specification in the AAS is not yet fully specified, and a liaison between Plattform Industrie 4.0 and IDSA was formed to solve the issue. In this context, the IDS Industrial Community was formed [34]. We, therefore, propose to use dataspace connectors across company borders and DTs inside the company [35].

#### 3.2.3. DT Registry and IDS Broker

In both DT and the data space domain, there are software components enabling the discovery and publication of data sources. In the case of the IDS, an IDS broker provides a list of IDS connectors with their available data offerings [4]. However, the broker only understands the IDS information model and cannot resolve DT requests, such as the AAS queries found in the AAS registry API.

It is vital for participants to register their connector in a broker, since the participants in a data space might not know each other before data transfer. Likewise, it is vital to register the DT in a registry so that other DTs can find and interact with each other.

In the AAS specification, a component called the AAS registry is used to keep track of all registered AAS instances [3]. Like the IDS broker, the AAS registry provides a list of available AASs and their submodels.

The data exchange API building block also includes registry mechanisms in their specification; for example, IDS communication includes IDS broker registration. The brokers only store metadata about the data offering, while the data reside inside the company. As an example, brokers such as the EDC federated catalogue or IDS metadata broker can be used to store connector descriptions [12,36].

It is also vital to enable data space participants to find specific DT offerings, such as a DT describing a certain asset aspect. For this, we propose to include AAS identifiers in the data space catalogue description, which in turn allows participants to query the IDS broker for participants offering AASs.

In a previous project [37], we combined the functionality of IDS brokers and AAS registries in a single component. This allowed customers to make use of the AAS API to look for available AASs even if the provider AAS was protected by IDS connectors. This made sense in cross-company scenarios where the AAS needs to find and interact with other AASs while not being aware of IDS connectors while leveraging data sovereign data transfers. The task of finding other AASs to communicate with was then handled by the consumer AAS or external systems using the AAS API.

However, the data transfers are still required to happen between the connectors so that a valid usage contract can be negotiated. This means that the customer AAS or an external system still needs a connector to handle the data transfer.

The necessity for such a combined broker/registry in I4.0 use cases depends on the requirements, more specifically on whether the customer AAS is tasked with finding the correct data providers. We currently assume that a human operator browses the IDS broker listing AAS data sources, selects and negotiates the data usage, and initiates the data transfers to the customer AAS.

#### 3.2.4. DT Semantics and IDS Vocabulary Provider

Semantic meaning in DTs refers to the way the information is structured, communicated, and understood within the DT and the application domain. It involves defining a standardized way to represent and communicate asset-related information. This standardization is crucial for ensuring interoperability among different devices, systems, and services.

In the context of the AAS, there are several concepts to establish syntactic and semantic interoperability. Semantic IDs are used to link a semantic specification to a submodel or submodel element. This specification provides meaning to the element, for example, an ontology or a formal specification describing the element in detail [3]. It is possible to define own dictionaries in the AAS that contain semantic definitions of the submodel elements. These semantic definitions are called concept descriptions and are mainly used for attributes and data types in the AAS [38].

While the meta model of the AAS creates a rough syntactic structure, the aspects of an asset can be split across several submodels and elements in any way the company sees fit. Submodel templates define the aspects of the asset to be represented in a specific structure and with specific elements. They are created and standardized by Industry 4.0 working groups allowing for better interoperability between different systems and devices, if companies implement them [39].

In data spaces, semantics can also be described in the metadata of the offered data resources. In order to resolve the semantic description, the IDS vocabulary provider is a special connector that can be queried for the required semantic description [40]. It can host several vocabularies and ontologies used in annotations and descriptions of data.

In a previous project [37], we tried to leverage this component to add missing semantic IDs in an AAS. Since the IDS vocabulary provider hosts several vocabularies and ontologies, it can be queried when an AAS is shared over a connector. For example, an AAS with an element “tire” is shared without describing it with a semantic ID. In this case, the IDS vocabulary provider can be queried to find a suitable semantic ID according to the context of the AAS. The context might be the asset of the AAS or surrounding elements which are then used to find a suitable vocabulary or ontology. Following the “tire” example, the asset might be of type “rail-borne vehicle”, allowing to resolve into eClass vocabulary “28-04-07-07 wheel tire”. The approach can include simple string or AI-supported matching. This matching can happen before the data are offered or during data transit in the data space. However, we strongly suggest making use of submodel templates and semantic IDs before the AAS is shared to enable browsing for suitable data resources.

After the semantic IDs are used to reference the ontologies, the ontology must be available for all participants to resolve the semantic meaning. This can be achieved by referencing a URI [40] publicly available or hosted by a vocabulary provider.

## 4. Our Approach for Integration between AAS and IDS

In this section, we present our approach of AAS integration into the IDS, based on the previously discussed components and design possibilities.

### 4.1. Simplified High-Level Architecture with Minimal Set of Building Blocks

**Figure 5:**
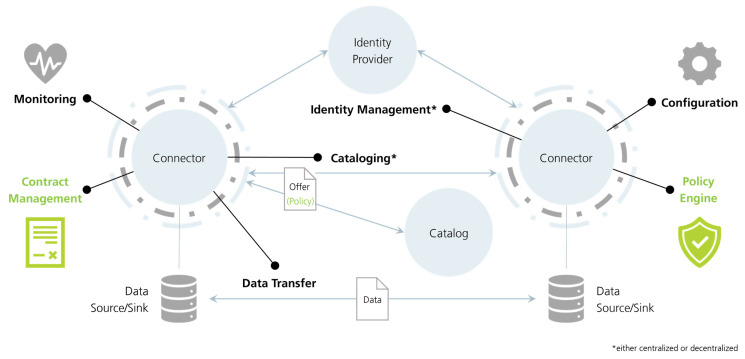
Connector in data space by Eclipse Foundation. Reproduced with permission from [32].

While the data space architecture shown in Section 3.1 includes nearly all components a data space has to offer, they are not necessarily required for the operation of a minimal data space. Figure 5 shows a data space architecture by the Eclipse Foundation which focuses solely on the necessary components [32]. A minimal data space usually only requires connectors and identity providers. For example, in a small data space for Industry 4.0 use cases, participants browse the data catalogues of other participants by interacting with the data provider connector. Without a broker, the participants must know each other before data transfers and agree on the connectors and identities to be used.

If the DT integration of Figure 4 is combined with Figure 5, we can leverage the AAS as a data source/sink with several open-source implementations of connector, identity providers, and DT tools in a data space. Table 2 shows the proposed software components and functions they provide.

**Figure 6:**
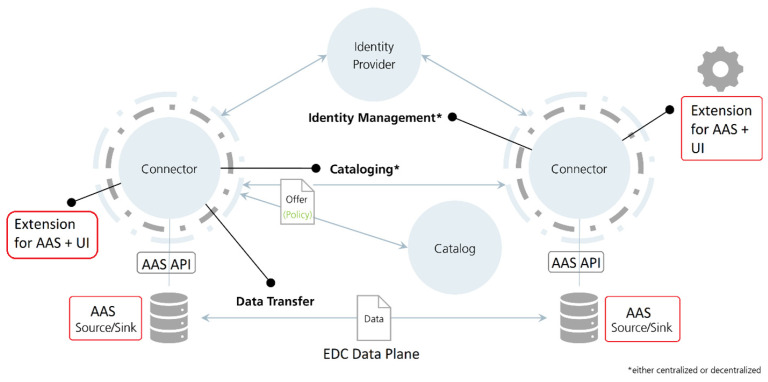
Minimal data space adapted for AAS. Reproduced with permission from [32].

The resulting architecture we propose and implement with these components is shown in Figure 6. The identity provider can also be realized by components such as the Omejdn DAPS or EDC Identity Hub.

As previously mentioned, to share data in a data space, a description of data sources in the connector catalogue is required. This is mostly a manual process where the data to be shared are selected, described, and fitted with a usage contract. In the case of the AAS as a data source, each submodel or element requires such a description. Since huge models can comprise hundreds of submodels and thousands of elements, manual processing is only feasible during early development stages.

**Figure 7:**
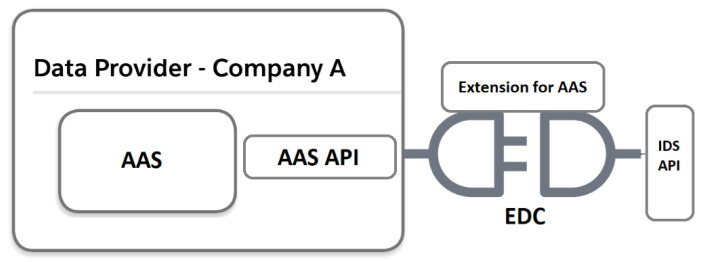
EDC Extension for AAS integrating AAS into data space.

Since the EDC can be extended with “EDC Extensions”, we created a new component “EDC Extension for AAS” to simplify this process for AAS providers and consumer. We made the code available open source to support other projects applying AAS in IDS [41]. The extension provides functionality for providers sharing AAS and consumers interacting with AAS. Figure 7 shows the integration of the EDC Extension into EDC and the AAS as a data source.

Data providers can upload their AAS JSON/AASX file or fill in the URL to automatically share the AAS over the EDC. When changes are made to the AAS of a product or process, the extension automatically updates the corresponding resources in the data space. Depending on the use case, changes can happen frequently, in which case manual processing is not feasible and the extension is needed.

On the consumer side, we also simplify the request of AAS data and provide a user interface for both provider and consumer. Previously, the consumer would have to send several EDC requests to the provider to request and accept the usage contract, as well as initiate the data transfer. The GUI now handles the negotiation and informs, if necessary, the consumer about new contracts. After accepting the contract, the data transfer is started. With the GUI, consumers can graphically browse the data catalogue of other connectors and select suitable AAS data sources.

### 4.2. Architecture and Modules of the EDC Extension for AAS

**Figure 8:**
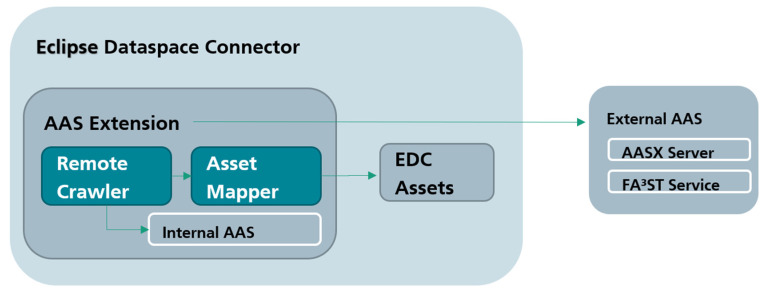
EDC Extension for AAS (provider view).

In Figure 8, the architecture of the EDC extension is shown from a provider perspective. Main considerations when designing the extension include the following:

- Supporting all AAS files/formats;
- Supporting different implementations of AAS services, if the AAS is already in a running service;
- Providing graphical interface;
- Reducing user effort.

We differentiate between already running AASs (external) and AASs that will be started from a model file (internal). In the first case, the AAS is already running as a service in the company, for example, with implementations such as NOVAAS [26] or FA³ST Service [25]. In this case, the URL of the AAS must be provided so that all resources can be created automatically on the basis of external AAS.

The remote crawler connects to the external AAS service and tries to retrieve all AASs found in the service. For this, the remote crawler uses the Eclipse AAS4J [44] library to support different serializations of the AAS such as JSON and XML. We are currently in the process of updating the remote crawler to support the newest version of AAS4J. Additionally, the AAS4J library provides serializers and deserializers, which are useful to deliver relevant parts of the model to the asset mapper. The asset mapper is responsible for mapping each AAS submodel and element into a newly created EDC Asset. For this, the asset mapper directly accesses the asset index of the EDC. Each submodel and element is then mapped into an EDC asset. In theory, the extension could be decoupled from the EDC by interacting with the EDC API to create assets and contracts, but the EDC provides convenient access to several internal components for extensions.

If the extension instead receives an AAS model file, the remote crawler starts a FA³ST Service internally by handing over the file. We created an interface, which can be implemented for several different AAS services. Currently, the interface is only implemented for FA³ST Service.

Initially, a default usage contract can be chosen, which the asset mapper applies to all elements to simplify the sharing process. Later, the contract can be modified for each EDC resource in the user interface. Listing 1 shows our default policy, which simply grants usage permission without constraints and is included in our default contract.
**Listing 1:** EDC policy.1 [ 2  { 3   “id”: “123”, 4   “policy”: { 5     “permissions”: [ 6      { 7       “edctype”: “dataspaceconnector:permission”, 8       “action”: { 9         “type”: “USE”, 10          “includedIn”: null, 11          “constraint”: null 12         }, 13         “constraints”: [], 14         “duties”: [] 15       } 16     ], 17     “prohibitions”: [], 18     “obligations”: [], 19     “extensibleProperties”: {} 20   }, 21   “asset”: {} 22   } 23 ]

**Figure 9:**
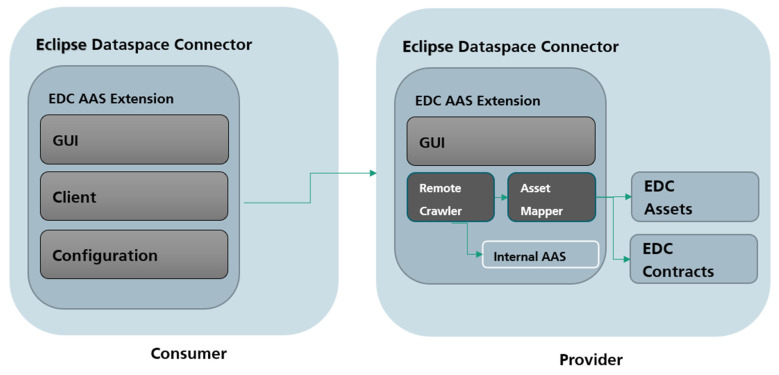
EDC Extension for AAS (provider and consumer).

On the consumer side, the EDC Extension for the AAS provides GUI, client, and configuration modules. The GUI module is a web-based service to simplify interaction with the client and configuration modules. The configuration module allows consumers and providers to make changes to their EDC without editing configuration files. For example, several EDC settings and configurations of the default contract can be changed here. The client module is a simple EDC client to simplify the AAS data request for consumers. For this, it bundles several API calls and lets the consumer decide if new contracts should be accepted. Figure 9 shows the complete view with interactions between provider and consumer. It should be noted that the EDC extension always contains all modules of both provider and consumer, but only the relevant modules for each are shown in Figure 9. In conjunction, a consumer can, at any time, become a provider and provide AASs while consuming AAS data.

### 4.3. Example of AAS in IDS in PCF Use Case

**Figure 10:**
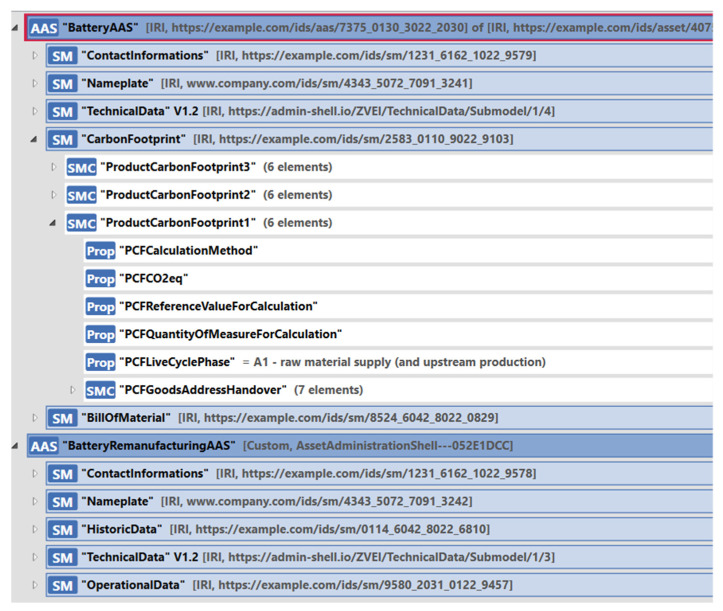
AAS example with PCF.

In this section, we go into more detail on how our approach can be applied in a concrete use case. For this, we want to model an AAS for a car battery and an AAS for the disassembly of the battery. As a complete DPP for the battery is still a work in progress, we modeled only some key aspects with AAS submodels. We introduced the recently released product carbon footprint (PCF) [39] submodel template into the model to describe the carbon footprint of the battery inside the AAS. We also used the digital nameplate template to provide identifying information about the battery. For other submodels, we created our own templates, which will be replaced in the future. The AASs are shown in the AASX Package Explorer tool [45] in Figure 10.

To synchronize the AAS with the battery and disassembly process, our AAS implementation FA³ST service [25] offers the concept of “asset connections”; for example, synchronization of process data via OPC UA servers or connections to data sources via HTTPS are possible with simple configuration of the service. We actively try to improve all aspects of the “asset connection” concept to support Industry 4.0 use cases in implementing DTs and data integration.

In our lab, we installed several power and air pressure sensors to simulate sensor data in disassembly processes. These sensors are connected to a Siemens PLC and are, in our case, available via the built-in Siemens OPC UA sever. Assuming that the disassembly requires power (power tools) or compressed air (pneumatic impact wrenches), we simulated a CO_2_ equivalent for testing purposes based on the German energy mix which includes fossil fuels. On the basis of the process duration, this CO_2_ equivalent is accumulated and stored in the disassembly process AAS. For this, we deployed the FA³ST service and loaded our previously created models. The FA³ST service was configured to connect to the sensors in our lab and store this CO_2_ equivalent.

**Figure 11:**
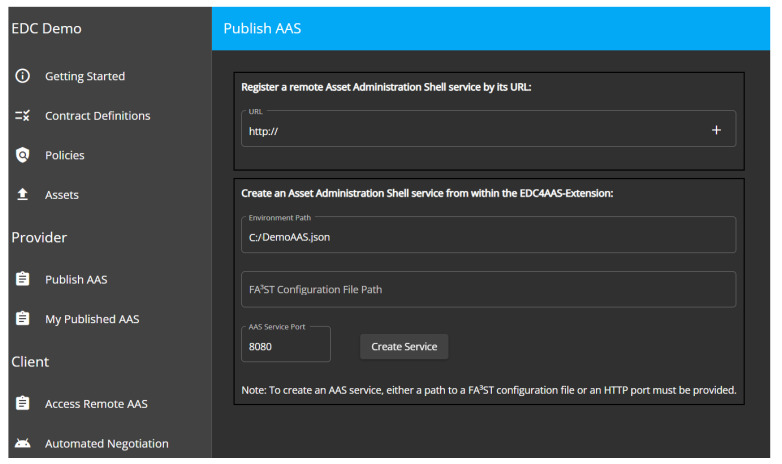
EDC Extension for AAS—provider GUI.

The next steps involve the sharing of the AASs in a data space. For this, we deployed the EDC containing our EDC Extension with Java in the lab. We inserted the link to the AAS (provided by FA³ST service) in the extension GUI, such that it would be automatically shared over the EDC. A default contract was chosen to be used as a usage contract for all elements. Figure 11 shows the GUI to select the AAS to be shared.

**Figure 12:**
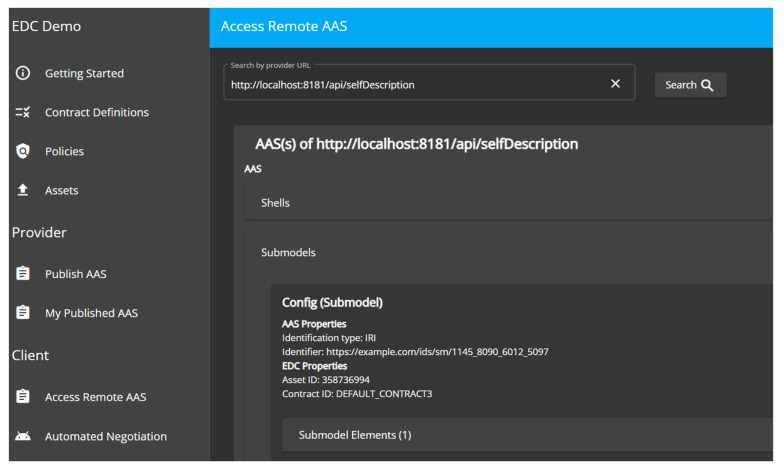
EDC Extension for AAS—consumer UI.

Figure 12 shows a consumer browsing and selecting AAS data sources provided by another EDC.

For identity management, we used the Omejdn DAPS identity provider [42]. A certificate was generated for our EDC by this DAPS. A broker was not set up in the lab, but publicly available IDS brokers can be used in conjunction with a broker extension for the EDC [46].

As previously stated, our next goal is the modeling of a complete DPP within an AAS and improving asset connectivity. Furthermore, the AAS in general requires more submodel templates to represent common assets and DPPs, which must be developed jointly with the IDTA. Additionally, data processing, e.g., AI models, must be integrated into the AAS and the data space.

Unfortunately, components such as the EDC Extension for AAS must be created for other IDS connectors, and incompatibility between IDS connectors must be solved on both a design and an implementation level.

All AAS implementations (including the FA³ST service) must be updated to the new AAS model and API version 3.0, which was released quite recently.

## 5. Comparison with State of the Art

In related papers, the AAS meta-model serves as a common data model, and IDS facilitates secure communication and integration among different AAS-based systems. The papers differ mainly in the following aspects:

- The types of IDS connectors considered,
- The generalization of the approach to use any implementation of the AAS specification,
- Supporting an AAS provider and an AAS consumer in integrating an AAS with an IDS connection in an automated way.

The Catena-X approach [47] is quite complementary to ours as they also consider the EDC connector and support any AAS. However, while they create an EDC asset on a submodel level, our approach allows the automatic creation of an EDC asset for each submodel element regardless of the nesting level. In addition, we provide a tool to help create these numerous EDC assets. Thus, our tool is also beneficial in Catena-X. The other distinction is that the Catena-X approach is based on a DT registry where AAS descriptors are stored. These descriptors also contain an EDC endpoint that points to the EDC endpoint in the corresponding EDC. Our approach favors an IDS broker to discover the consumer and provider EDC with AASs. This means that we clearly split between the IDS-RAM and the AAS specification in order not to introduce domain-specific extensions to AAS components or vice versa.

Eclipse BaSyx [48] is integrated with the Advaneo Trusted Data Hub [49]. A toolchain was developed that enables factories to use machine learning models and to analyze data collaboratively. Eclipse BaSyx with its support for AAS provides the raw data of the factory. Currently, the interface between an AAS and an IDS connector is based only on the MQTT protocol. This means that part 2 of the AAS specification is not fully implemented (e.g., HTTP via REST APIs is not supported), which is a differentiation from our solution. While the proposed approach is based on the trusted connector, we also started our work with the base connector, which was later extended with the trusted connector and the dataspace connector (DSC). We currently only support the EDC connector as the DSC was superseded by it, and the EDC is the most used since it has a sizable open-source community. An overview of the IDS connectors can be found in [50]. Furthermore, while the proposed approach is based on the BaSyx implementation of the AAS specification, our approach not only supports the FA³ST implementation of AAS but is designed to be open to any AAS implementation that supports version 3.0 of the metamodel.

In [51], the authors proposed a manufacturing-as-a-service system for the execution of remote production orders built upon the integration of AAS capabilities and IDS connectors. Unlike our solution, the approach is based on dataspace connectors that require other methods for creating IDS resources. However, there is no support to create them automatically to help integrate the two technologies in a convenient way. Additionally, the authors do not share the AASs directly with the IDS connector, but an AAS catalogue of the assets. The AAS catalogue is the list of all available AAS inside the AAS registry as defined in the AAS specification. This means that only the submodel level data are stored there; therefore, there is no support for sharing the whole AAS. In their architecture, an additional orchestrator is used to find communication information of the asset in the catalogue to contact it directly for data elements and values through the orchestrator.

In [35], the authors described how Industry 4.0 and IDS concepts and tools can be combined to support the creation of digital twins according to the architectural framework described in ISO DIS 23247. We followed this approach and proposed a concrete technical solution for the realization with a connector extension similar to an IDS app specialized for integration with an AAS.

In [52], several possibilities for integrating an AAS into the IDS architecture were discussed. The authors proposed an IDS app that is specifically tailored to easily connect AAS to IDS. In this case, an AAS is considered as an external IDS resource, and critical data are processed by IDS data applications. The proposed approach is based on FA³ST and dataspace connectors.

In this paper, we extended the proposed approach not only by abstracting from the concrete AAS implementation and replacing the dataspace connector with the EDC connector, but more importantly by providing support to users who are not familiar with all the details of AAS and EDC to integrate them in an effortless way.

## 6. Conclusions and Future Work

In this paper, we discussed digital twins in data spaces, more specifically the integration of the AAS concept into the IDS. We presented our approach of the integration and the implementations of the required components. As most data space building blocks are designed in a domain-agnostic way, reference data space implementations can be reused. The changes for digital twins focus on the data modeling of aspects such as carbon footprints or product passports. Furthermore, the automated integration of these data into the data space with additional services or marketplaces following the principle of manufacturing as a service (MaaS) is an area of particular interest, which sets different domain data spaces apart. We propose the modeling of process and product data with AAS submodel templates or referencing established ontologies or vocabularies within the AAS. For the registration of these data in the data space, we implemented an EDC extension for the AAS to register AAS services as EDC resources. Similar extensions can be written for other IDS connectors. The extension must be updated continuously to support new versions of the AAS specification and the EDC. With the imminent introduction of security aspects into the AAS, the extension and the data space architecture must also reflect and incorporate these aspects; for example, access control in the AAS should not lock out the remote crawler of the extension.

The EDC extension for AAS requires further evaluation and will be improved with additional user-friendliness as required in future use cases. Additionally, more aspects of the products and processes need to be modeled in the AAS with standardized submodel templates. We will continue our involvement in IDTA working groups to specify these templates.

## Figures and Tables

**Table 1:** Data space components mapped to building blocks.

IDS Components/ Data Space Technical Building Blocks	Interoperability	Trust	Data Value
Connector	Provides data exchange API, stores provenance logs	Enables trusted exchange	Stores metadata and logs data usage
Identity provider		Provides identity management, enables trusted exchange	
Broker			Stores metadata, enables discovery and publication
Vocabulary provider	Stores data models		
Clearing house	Enables provenance and traceability		Enables data usage accounting
App store			Offers additional services
Marketplace			Offers additional services

**Table 2:** Software components in minimal data space.

Component	Function	Link
FA³ST Service	Provides an execution environment for the AAS	[25]
Eclipse Dataspace Connector (EDC)	IDS connector for data exchange between DS participants	[32]
EDC Extension for AAS	Integrates the AAS into the EDC and simplifies AAS usage for providers and consumers	[41]
Omejdn DAPS	IDS identity provider (option 1)	[42]
EDC Identity Hub	IDS identity provider (option 2)	[43]

## Data Availability

Not applicable.
